# Shifts in the Gut Microbiota Composition Due to Depleted Bone Marrow Beta Adrenergic Signaling Are Associated with Suppressed Inflammatory Transcriptional Networks in the Mouse Colon

**DOI:** 10.3389/fphys.2017.00220

**Published:** 2017-04-12

**Authors:** Tao Yang, Niousha Ahmari, Jordan T. Schmidt, Ty Redler, Rebeca Arocha, Kevin Pacholec, Kacy L. Magee, Wendi Malphurs, Jennifer L. Owen, Gregory A. Krane, Eric Li, Gary P. Wang, Thomas W. Vickroy, Mohan K. Raizada, Christopher J. Martyniuk, Jasenka Zubcevic

**Affiliations:** ^1^Department of Physiological Sciences, College of Veterinary Medicine, University of Florida (UF)Gainesville, FL, USA; ^2^Cellular and Molecular Pathology Branch, National Toxicology Program, National Institute of Environmental Health Sciences, Research Triangle ParkDurham, NC, USA; ^3^Division of Infectious Diseases and Global Medicine, Department of Medicine, University of FloridaGainesville, FL, USA; ^4^Department of Physiology and Functional Genomics, College of Medicine, University of FloridaGainesville, FL, USA

**Keywords:** gut microbiota, immune cells, sympathetic nervous drive, bone marrow, colon

## Abstract

The brain-gut axis plays a critical role in the regulation of different diseases, many of which are characterized by sympathetic dysregulation. However, a direct link between sympathetic dysregulation and gut dysbiosis remains to be illustrated. Bone marrow (BM)-derived immune cells continuously interact with the gut microbiota to maintain homeostasis in the host. Their function is largely dependent upon the sympathetic nervous system acting via adrenergic receptors present on the BM immune cells. In this study, we utilized a novel chimera mouse that lacks the expression of BM beta1/2 adrenergic receptors (b1/2-ARs) to investigate the role of the sympathetic drive to the BM in gut and microbiota homeostasis. Fecal analyses demonstrated a shift from a dominance of Firmicutes to Bacteroidetes phylum in the b1/2-ARs KO chimera, resulting in a reduction in Firmicutes/Bacteroidetes ratio. Meanwhile, a significant reduction in Proteobacteria phylum was determined. No changes in the abundance of acetate-, butyrate-, and lactate-producing bacteria, and colon pathology were observed in the b1/2-ARs KO chimera. Transcriptomic profiling in colon identified Killer Cell Lectin-Like Receptor Subfamily D, Member 1 (Klrd1), Membrane-Spanning 4-Domains Subfamily A Member 4A (Ms4a4b), and Casein Kinase 2 Alpha Prime Polypeptide (Csnk2a2) as main transcripts associated with the microbiota shifts in the b1/2-ARs KO chimera. Suppression of leukocyte-related transcriptome networks (i.e., function, differentiation, migration), classical compliment pathway, and networks associated with intestinal function, barrier integrity, and excretion was also observed in the colon of the KO chimera. Moreover, reduced expression of transcriptional networks related to intestinal diseases (i.e., ileitis, enteritis, inflammatory lesions, and stress) was noted. The observed suppressed transcriptome networks were associated with a reduction in NK cells, macrophages, and CD4^+^ T cells in the b1/2-ARs KO chimera colon. Thus, sympathetic regulation of BM-derived immune cells plays a significant role in modifying inflammatory networks in the colon and the gut microbiota composition. To our knowledge, this study is the first to suggest a key role of BM b1/2-ARs signaling in host-microbiota interactions, and reveals specific molecular mechanisms that may lead to generation of novel anti-inflammatory treatments for many immune and autonomic diseases as well as gut dysbiosis across the board.

## Introduction

The gut harbors trillions of bacteria that modulates the host homeostasis within and outside the intestinal tract. Recently, emerging evidence has demonstrated the association between gut dysbiosis and neurogenic diseases and conditions, such as hypertension (Mell et al., 2015; Yang et al., 2015), sleep apnea (Durgan et al., 2016), and anxiety (Cryan and Dinan, 2012), all marked by significantly altered sympathetic activity. However, the bidirectional communication between the brain and gut microbiota presents us with difficulties in determining the cause-effect relationship in disease, which subsequently hinders the efficient treatment.

The sympathetic nervous system (SNS) and the immune system (IS) are reciprocally regulated to maintain physiologic homeostasis and defend the host from pathologic threats (Bellinger and Lorton, 2014; Jänig, 2014). Although both alpha (a-) and beta-adrenergic receptors (b-ARs) are expressed on immune cells (ICs) (Jänig, 2014; Lorton and Bellinger, 2015), it has been demonstrated that the adaptive IS responds to SNS cues predominantly via the b2-ARs (Lorton and Bellinger, 2015), and that stimulation of b2-ARs on ICs modulates lymphocyte development, survival, proliferation, activation, and trafficking (Jänig, 2014; Lorton and Bellinger, 2015). On the other hand, the gut, as the largest immune organ in the body, reciprocally communicates with the host IS, and a variety of intestinal diseases have been associated with dysregulation of the IS [e.g., inflammatory bowel diseases (Baumgart and Carding, 2007; Degruttola et al., 2016) and colorectal cancer (Gao et al., 2015)]. Exogenous factors (i.e., pathogen infection, diet, physical exercise.), that have been demonstrated to alter the gut microbiota can also exhibit profound effects on the IS (Karczewski et al., 2014; Lightfoot et al., 2014; Petriz et al., 2014; Vieira et al., 2014). In addition, several extra-intestinal autoimmune diseases [i.e., multiple sclerosis, arthritis, and type 1 diabetes (Kamada et al., 2013)] have been associated with gut dysbiosis. Typically, one consequent feature of the gut dysbiosis is the reduced production of short chain fatty acids (SCFAs), the beneficial metabolites derived from anearobic fermentation of gut microbiota. Therefore, evaluation of SCFAs level become a reliable indicator of gut homeostasis (Harris et al., 2012).

Collectively, the critical regulatory role of the SNS in the control of IS, and the importance of the IS in maintaining gut homeostasis, led to our hypothesis that the interaction between the SNS and the gut microbiota involves SNS-mediated modulation of IS function. This may be particularly relevant in neurogenic diseases, due to its association with both over-activated IS and gut dysbiosis. Given that the BM is the origin of ICs, and that the proinflammatory BM contributes to the modulation of neuroinflammation and autonomic functions (Santisteban et al., 2015), we postulated that manipulation of the SNS effects on the BM ICs will in turn affect the host gut microbiota composition that is associated with alterations in the gut IS. To test this hypothesis, we utilized the BM b1/2-ARs KO chimera mice, a model in which the irradiation-depleted wild type (WT) BM is reconstituted with BM from the b1/2 ARs KO mice (Ahmari et al., 2016). This manipulation results in diminished effects of SNS on the BM ICs, thus allowing investigation of its effects on the gut microbiota composition. Herein, we demonstrate that loss of BM b1/2-ARs signaling results in shifts in the gut microbiota composition and suppression of colon IS, indicating a novel mechanistic association between the host BM SNS, IS, and the gut microbiota. This is highly significant considering that many dysbiotic and immune conditions are also associated with dysregulation of the SNS (Cryan and Dinan, 2012; Bellinger and Lorton, 2014; Bienenstock et al., 2015).

## Materials and methods

### Animals

Male chimera mice were generated and validated as previously described (Ahmari et al., 2016). Control chimera mice (Control) were generated by reconstitution of WT C57BL/6J whole BM cells into sublethally irradiated age- and sex-matched C57BL/6J mice by comparable irradiation and reconstitution protocols. Following irradiation, all chimera mice were treated with 0.4% Baytril antibiotic (enrofloxacin; Bayer) in drinking water, and were given moist chow and Nutrical supplement daily during the first 3 weeks. Following this, the mice were allowed to recover for 9 weeks before fecal collections. All experimental procedures were completed in accordance with the approved protocols by the University of Florida Institute for Animal Care and Use Committee, and complied with the standards stated in the National Institutes of Health's Guide for the Care and Use of Laboratory Animals. All mice were housed in a temperature-controlled room (22°–23°C) on a 12:12 h light-dark cycle, in specific pathogen-free cages, and had access to standard mouse chow and water *ad libitum*.

### Microbiota sequencing and analyses

Fecal samples were collected from C57 chimera and b1/2 ARs KO chimera mice 3 months post-irradiation and BM reconstitution, to allow for full recovery of the mice, as previously described (Ahmari et al., 2016). The analyses of microbial composition were performed, as previously described (Yang et al., 2015). Briefly, fecal DNA (*N* = 6/group) was extracted by using ZR fecal DNA MiniPrep (Zymo Research, Irvine, CA). Bacterial 16S rRNA containing V4-V5 regions were obtained using Illumina Miseq compatible primers. PCR amplicons were purified (Qiagen, Madison, WI) following separation on an agarose gel, and quantified by the Qubit Fluorometer (Invitrogen, Grand Island, NY). Equal amounts of amplicon for each sample were pooled and qualified with Kapa SYBR fast qPCR kit (Kapa Biosystems, Inc., Woburn, MA). Pooled samples were run on the Illumina Miseq platform using Miseq v2 reagent kit (Illumina, Inc., San Diego, CA). Primers used for amplification are listed in Table 1.

**Table 1:** **Primer sequences for microbiota 16S V4–5 amplification**.

**Name**	**Forward primer**	**Reverse primer**
**C1**	AATGATACGGCGACCACCGAGATCTACACTCTTTCCCTACACGA CGCTCTTCCGATCTTAGCTGCCAGCMGCCGCGGTAAT	CAAGCAGAAGACGGCATACGAGATCGGTCTCGGCATTCCTGCTGAA CCGCTCTTCCGATCTGTAGCACCGTCAATTYYTTTRAGTTT
**C2**	AATGATACGGCGACCACCGAGATCTACACTCTTTCCCTACACGAC GCTCTTCCGATCTAGCTACCCAGCMGCCGCGGTAAT	CAAGCAGAAGACGGCATACGAGATCGGTCTCGGCATTCCTGCTGAACCG CTCTTCCGATCTGTAGCACCGTCAATTYYTTTRAGTTT
**C3**	AATGATACGGCGACCACCGAGATCTACACTCTTTCCCTACACGAC GCTCTTCCGATCTAGCTACCCAGCMGCCGCGGTAAT	CAAGCAGAAGACGGCATACGAGATCGGTCTCGGCATTCCTGCTGAA CCGCTCTTCCGATCTCACTGTCCGTCAATTYYTTTRAGTTT
**C4**	AATGATACGGCGACCACCGAGATCTACACTCTTTCCCTAC ACGACGCTCTTCCGATCTAGCTACCCAGCMGCCGCGGTAAT	CAAGCAGAAGACGGCATACGAGATCGGTCTCGGCATTCCTGCTG AACCGCTCTTCCGATCTATTGGCCCGTCAATTYYTTTRAGTTT
**C6**	AATGATACGGCGACCACCGAGATCTACACTCTTTCCCTAC ACGACGCTCTTCCGATCTAGCTACCCAGCMGCCGCGGTAAT	CAAGCAGAAGACGGCATACGAGATCGGTCTCGGCATTCCTGCTGAACC GCTCTTCCGATCTGATCTGCCGTCAATTYYTTTRAGTTT
**C7**	AATGATACGGCGACCACCGAGATCTACACTCTTTCCCTACACG ACGCTCTTCCGATCTAGCTACCCAGCMGCCGCGGTAAT	CAAGCAGAAGACGGCATACGAGATCGGTCTCGGCATTCCTGCTGAACC GCTCTTCCGATCTAAGCTACCGTCAATTYYTTTRAGTTT
**KO1**	AATGATACGGCGACCACCGAGATCTACACTCTTTCCCTAC ACGACGCTCTTCCGATCTTAGCTGCCAGCMGCCGCGGTAAT	CAAGCAGAAGACGGCATACGAGATCGGTCTCGGCATTCCTGCTGA ACCGCTCTTCCGATCTTACCATCCGTCAATTYYTTTRAGTTT
**KO2**	AATGATACGGCGACCACCGAGATCTACACTCTTTCCCTACA CGACGCTCTTCCGATCTAGCTACCCAGCMGCCGCGGTAAT	CAAGCAGAAGACGGCATACGAGATCGGTCTCGGCATTCCTGCTGAACC GCTCTTCCGATCTCGTGATCCGTCAATTYYTTTRAGTTT
**KO3**	AATGATACGGCGACCACCGAGATCTACACTCTTTCCCTACACG ACGCTCTTCCGATCTAGCTACCCAGCMGCCGCGGTAAT	CAAGCAGAAGACGGCATACGAGATCGGTCTCGGCATTCCTG CTGAACCGCTCTTCCGATCTACATCGCCGTCAATTYYTTTRAGTTT
**KO4**	AATGATACGGCGACCACCGAGATCTACACTCTTTCCCTACAC GACGCTCTTCCGATCTAGCTACCCAGCMGCCGCGGTAAT	CAAGCAGAAGACGGCATACGAGATCGGTCTCGGCATTCCTGCTGAA CCGCTCTTCCGATCTGCCTAACCGTCAATTYYTTTRAGTTT
**KO5**	AATGATACGGCGACCACCGAGATCTACACTCTTTCCCTA CACGACGCTCTTCCGATCTAGCTACCCAGCMGCCGCGGTAAT	CAAGCAGAAGACGGCATACGAGATCGGTCTCGGCATTCCTGCTGAACCG CTCTTCCGATCTTCAAGTCCGTCAATTYYTTTRAGTTT
**KO6**	AATGATACGGCGACCACCGAGATCTACACTCTTTCCCTACACGAC GCTCTTCCGATCTAGCTACCCAGCMGCCGCGGTAAT	CAAGCAGAAGACGGCATACGAGATCGGTCTCGGCATTCCTGCTG AACCGCTCTTCCGATCTCTGATACCGTCAATTYYTTTRAGTTT

The bioinformatics analyses were performed as described previously (Lightfoot et al., 2014; Yang et al., 2015). Briefly, sequenced reads were demultiplexed according to a combination of forward and reverse indices. Subsequently, obtained reads were filtered based on additional requirements: exact match to sequencing primers and quality score of 30 or more. Filtered reads were joined using FLASh (Fast Length Adjustment of Short reads) software, applied against Silva non-redundant 16S rRNA references database (v108), assigned taxonomic classifications at a 97% clustering threshold, and summarized in a refOTU table. Alpha, beta diversity, and principal coordinate analyses (PCoA) measures were created using custom R scripts and scripts from the QIIME package. Linear discriminant analysis (LDA) along with effect size measurements (LEfSe) in Galaxy was used to distinguish differentially significant features at each taxonomic level. Bacterial genus were identified based on 16S sequencing data and grouped based on the primary metabolite according to Bergey's manual as before described (Antharam et al., 2013; Yang et al., 2015).

### Short chain fatty acid (SCFA) extraction and high performance liquid chromatography (HPLC)

Fecal pellets (*N* = 4/group) were collected and SCFAs were extracted, as previously described (De Baere et al., 2013) with some modifications. Briefly, 200 μL of HCl was added to the fecal homogenates to preserve the volatile SCFAs, and vortexed vigorously to evenly suspend the fecal mass. Five milliliters of methylene chloride was used to extract the SCFAs with gentle rotation at room temperature for 20 min. After centrifugation at 3,500 rpm for 5 min, the supernatant was discarded. Five hundred microliters of 1N NaOH was added to the organic pellet, followed by an additional 20 min rotation at room temperature. After spinning at 3500 rpm for 5 min, the top aqueous phase was collected and mixed with 100 μL of HCl before filtered for injection. A 50 μL aliquot was used for each injection. A standard linear regression curve was generated for each of the acetate, lactate, propionate, and butyrate solutions separately, for calibration purposes. All of the standard solutions were extracted according to the method described above. Relative standard deviation (< 3.0%) was achieved by sequential injections of 10 mM standard mix (of all three SCFAs) into the mobile phase. The chromatographic separation was performed at room temperature using Perkin Elmer Series 200 HPLC System (Perkin Elmer Instruments, Norwalk, CT), equipped with an autosampler, quaternary pump, and 200 series UV/VIS detector. The analytical column used was Hypersil Gold aQ 150 × 4.6 mm 3 μm (Thermo Fisher Scientific, Walthan, MA). Samples were run in a combination of two solutions: 90% of mobile phase A 0.02 M phosphate buffer pH 2.2 and 10% of mobile phase B acetonitrile. Flow rate was set as 0.8 ml/min for 25 min. Wavelength used for detection was 210 nm.

### Tissue collection and isolation of total RNA for microarray analyses

Extraction of RNA from the distal colon was performed using 1 mL TRIzol® Reagent (Life Technologies, Carlsbad, CA), as per manufacturer's protocol. Final nucleic acid pellets were resuspended in 30 μL RNAse-DNAse free water. Total RNA integrity for all samples used in microarray and real-time PCR analyses was determined using the RNA 6000 Nano Assay Kit with the 2100 Bioanalyzer (Agilent Technologies, Santa Clara, CA). The mean RNA integrity value for all samples was 8.16 (*SD* ± 0.76).

### Microarray analyses and bioinformatics

The SurePrint G3 Mouse GE 8 × 60 K Microarray Kit (Agilent Product Number, G4852A Design ID 028005) was used for expression analyses (Agilent, Santa Clara, CA, USA). Sample sizes for microarray analysis were *N* = 7 per group. Briefly, the RNeasy Mini Kit was used to purify RNA prior to labeling, as per manufacturer's protocol (Qiagen, Valencia, CA, USA). RNA concentrations were determined using the NanoDrop-2000 spectrophotometer (Thermo Scientific). Microarray hybridizations were performed according to the One-Color Microarray-Based Gene Expression Analysis Low Input Quick Amp Labeling kit (Agilent V6.5, May 2010) and 10 ng total RNA per sample was used for labeling and hybridization as per our previous protocol (Feswick et al., 2014; Ornostay et al., 2016). Microarrays were scanned at 5 μm with the Agilent G2505 C Microarray Scanner, and Agilent Feature Extraction Software (v. 10.1.1.1) was used to extract raw signal intensities from microarray images. Microarray data were evaluated by manual inspection of the quality control parameters. All arrays were deemed high quality. Raw microarray data have been deposited into the NCBI Gene Expression Omnibus (GEO) database (Series GSE71632, GPL13912). Raw intensity data were imported into JMP® Genomics v7.0 (SAS Institute Inc., Cary, NC, USA). Intensity data were normalized using quantile normalization. Control probes were removed prior to identifying differentially expressed genes (DEGs), and the limit of detection was set to an intensity of 2.5 based on the Agilent spike-in controls. Therefore, any probe falling below this value was assigned a normalized intensity of 2.5. DEGs were identified using a one-way analysis of variance (ANOVA) followed by a false discovery rate (FDR) set at 5.0%. Cluster analyses were conducted in JMP Genomics v7.0 using probes that were differentially expressed (unadjusted *p* < 0.05). Two-way clustering using the Fast ward algorithm was conducted after each row was centered to a mean of zero (0) and variance scaled to one.

Pathway Studio 9.0 (Elsevier) and ResNet 10.0 were utilized for sub-network enrichment analysis (SNEA) of cell processes (Nikitin et al., 2003). The option of “best *P*-value, highest magnitude fold change” in Pathway Studio was used for duplicated probes. A total number of 32,734 mouse probes were successfully mapped to the program using the official gene name. SNEA was performed to identify transcriptome networks that were enriched in the distal colon. A Kolmogorov–Smirnov test with 500 permutations was conducted to determine whether networks were preferentially regulated compared to the background reference probability distribution. Networks were constructed based on common regulators of expression and regulators of specific cell processes. The enrichment *P*-value for a gene seed was set at *P* < 0.05. Additional details on the use of SNEA can be found in a previous publication (Langlois and Martyniuk, 2013).

### Real-time PCR validation

For distal colon microarray validation, primer sets from target genes were collected from primerbank (Table 2; (Wang et al., 2012)). Transcripts that showed dramatic and significant changes in expression levels were selected for microarray validation. The genes investigated included CFD, Cyp2e1, and Ms4a4b. *N* = 7–8/group. PCR was performed on CFX96 Touch™ Real-Time PCR Detection System (Bio-Rad, Hercules, California). The PCR conditions were as follows: 95°C for 3 min, followed by 40 cycles of 95°C for 30 s, primer annealing at 60°C for 30 s, and 72°C for 30 s. Dissociation curves were generated, starting at 65°C and ending at 95°C with increments of 0.5°C every 5 s. Gene expression was normalized to GAPDH and was determined using the relative ΔΔCt method based on the method described (Pfaffl, 2001). No reverse transcriptase control was used.

**Table 2:** **Primer sequences for microarray validation**.

**Name**	**Forward primer**	**Reverse primer**	**T_*m*_ (°C)**	**Size (bp)**
*Cfd*	CATGCTCGGCCCTACATGG	CACAGAGTCGTCATCCGTCAC	60	129
*Cyp2e1*	CGTTGCCTTGCTTGTCTGGA	AAGAAAGGAATTGGGAAAGGTCC	60	105
*Ms4a4b*	TGACACTTCAACCATTGCTACC	ACACATTTCCTGGAACATTGGTC	60	60

### Histopathologic evaluation

Colon tissue was fixed in 4% paraformaldehyde (PFA) and kept in 30% sucrose solution for 1 day. Tissue was then frozen at −80°C in O.C.T embedding, cross sectioned at 10 μm thickness via cryostat at −20°C, mounted onto glass slides, and routinely stained with hematoxylin and eosin (H&E). Sections from C57 (*N* = 2) and b1/2-ARs KO (*N* = 2) chimera mice were qualitatively evaluated for histopathologic lesions. Slides were digitally scanned using an Aperio System to produce representative images captured from the scans.

### Immunofluorescence

Colon tissues (*N* = 3/group) were fixed in 4% PFA, and kept in 30% sucrose solution for 1 day before freezing in O.C.T. Frozen tissues were sectioned at a thickness of 10 μm and mounted onto slides for immunofluorescent staining. Primary mouse anti-CD4 (1:500, #14-0041-85, eBioscience, San Diego, CA), anti-CD94 (1:100, #sc-390776, Santa Cruz Biotechnology, Dallas, Texas) and anti-CD11b (1:200, sc-20050, Santa Cruz Biotechnology, Dallas, Texas) were used, according to the manufacturer's protocol. Secondary antibody conjugated with Alexa Fluor 594 (Abcam, Cambridge, UK) was used for visualizing infiltration of CD4^+^ cells, NK cells, and macrophages, separately, in the intestines of both C57 and b1/2 ARs KO chimera. Three to four images/rat/antibody were used for quantification. Positive cell numbers were divided by total cell numbers, as indicated by DAPI staining.

## Results

### Alterations in the gut microbiota in b1/2-ARs KO chimera

**Figure 1:**
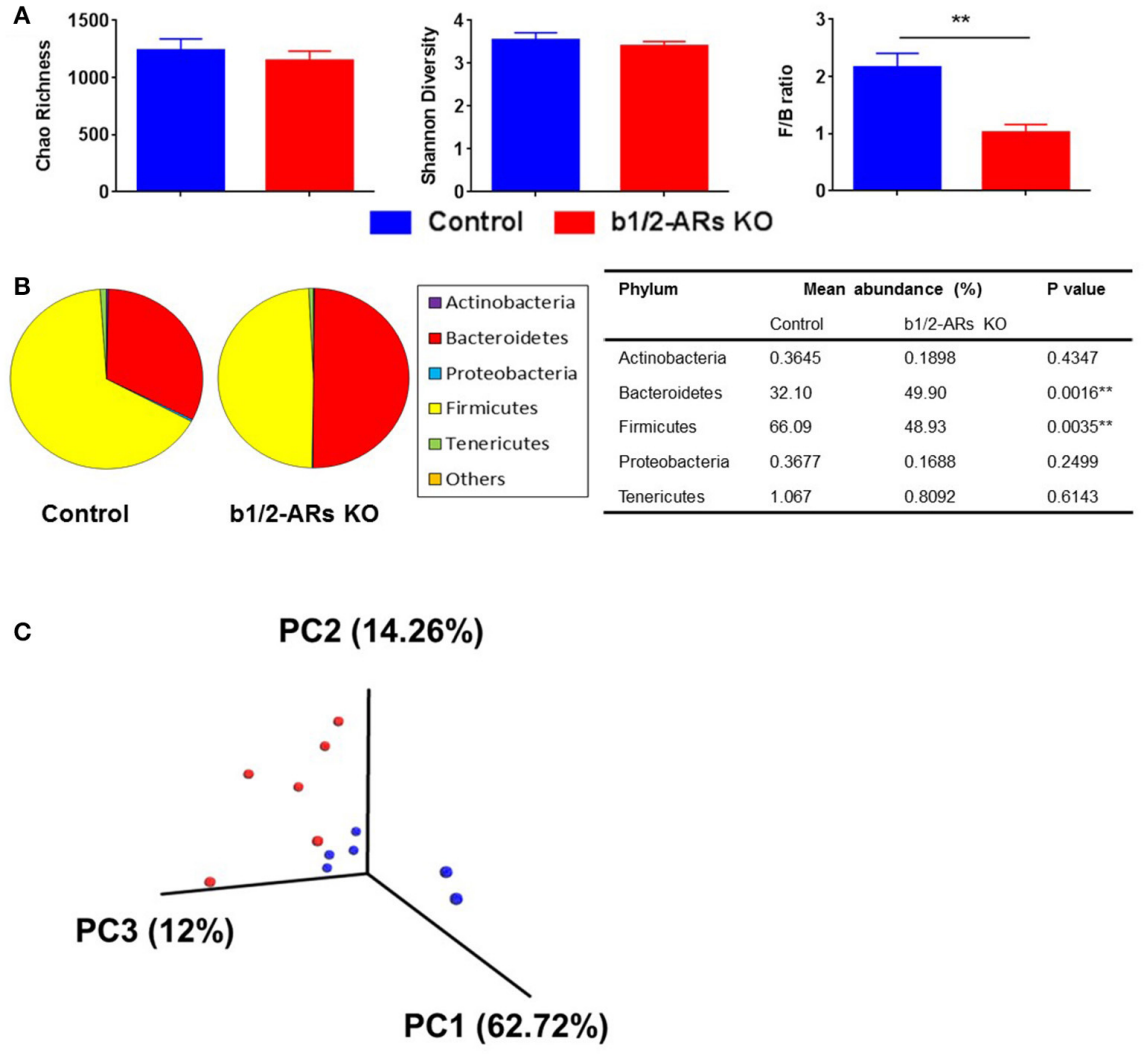
**Altered gut microbiota in the b1/2-ARs KO chimera. (A)** No significant difference in gut microbiota richness or diversity between the b1/2-ARs KO chimera and C57 control chimera. However, the Firmicutes to Bacteroidetes (F/B) ratio was significantly lower in the b1/2-ARs KO chimera compared to the C57 control chimera. **(B)** The decreased F/B ratio is attributed to the significant expansion of Bacteroidetes and contraction of Firmicutes. **(C)** Unweighted Principal coordinate analysis (PCoA) demonstrated a clear separation of red and blue clusters representing C57 and b1/2-ARs KO chimera groups, respectively. ^**^ indicates *P* < 0.01.

**Figure 2:**
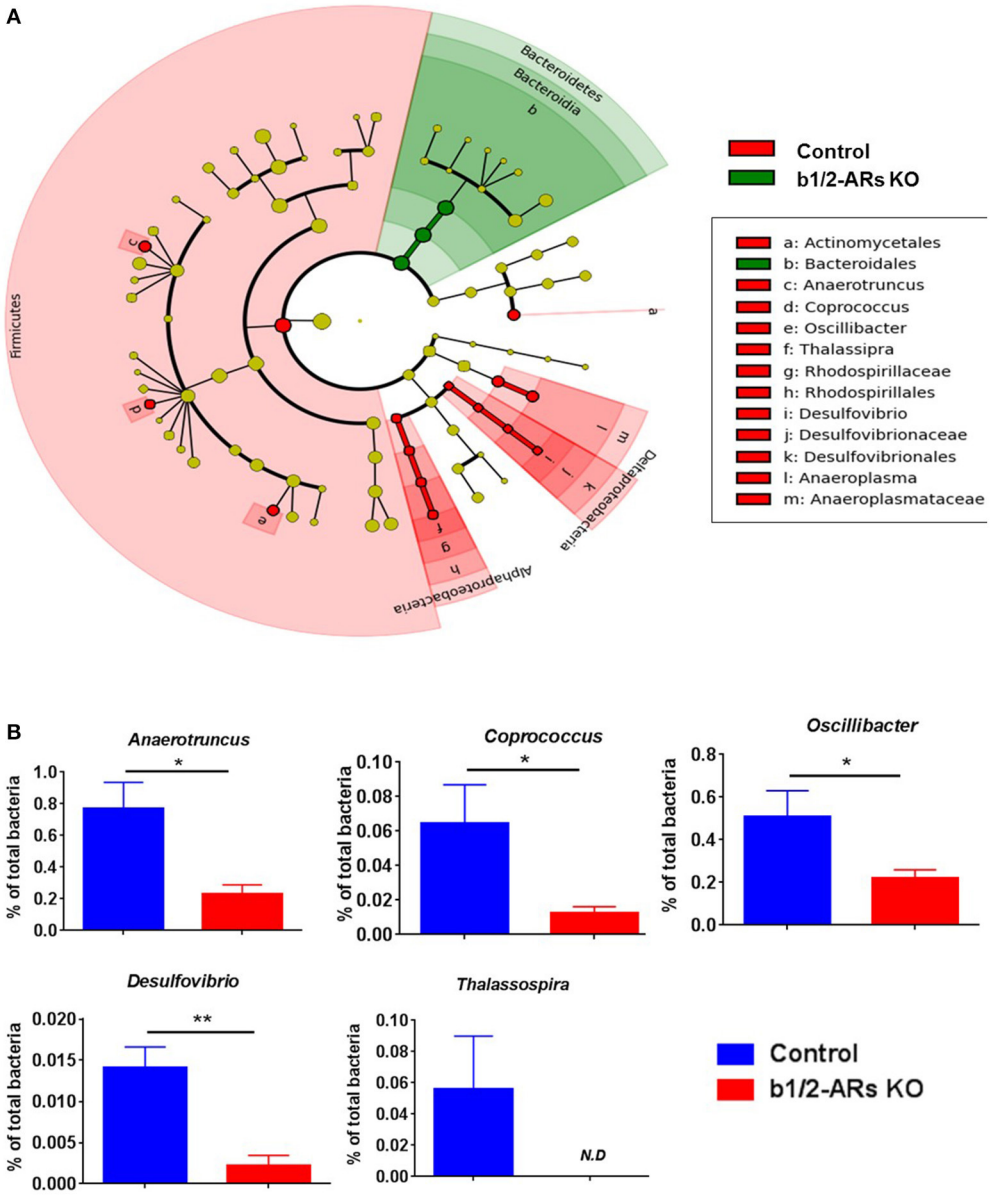
**Contraction of Proteobacteria phylum in the b1/2-ARs KO chimera. (A)** Taxonomic cladogram was generated by performing linear discriminant analysis (LDA) of effect size (LEfSe) in Galaxy (Segata et al., 2011). Each circle dot represents a bacterial taxon with its diameter proportional to the taxon's relative abundance. Differences are represented in colors where red indicates significant relative abundance in C57 chimera, green indicates significant relative abundance in b1/2-ARs KO chimera, and yellow indicates no significant differences. **(B)** Unpaired two-tailed *t*-test analyses of genera with significant differences between b1/2-ARs KO chimera and C57 control chimera. ^*^ indicates *P* < 0.05 and ^**^ indicates *P* < 0.01.

The rodent gut microbiota is generally dominated by Firmicutes and Bacteroidetes phyla, with Proteobacteria, Actinobacteria, and Tenericutes constituting the remaining phyla (Yang et al., 2013). Shifts in the bacterial proportions and phylotypes are widely used to evaluate gut homeostasis. Our 16S rRNA amplicon sequencing data suggested that Chao richness and Shannon diversity indices did not differ between C57 and b1/2-ARs KO chimeras (Figure 1A), indicating that deficiency in BM b1/2-ARs signaling did not induce changes in the microbiota species richness or biodiversity (Antharam et al., 2013; Yang et al., 2015; Mosca et al., 2016). However, a shift from a dominance of Firmicutes to Bacteroidetes, resulting in a reduction of the Firmicutes to Bacteroidetes (F/B) ratio, was observed (Figure 1A). We observed significant expansion of Bacteroidetes from approximate 1/3–1/2 of total bacteria reads and contraction of Firmicutes from approximate 2/3 to ½ of total bacteria reads in the b1/2-ARs KO chimera group (Figure 1B). No other change was observed at phylum level. Members of the alpha and delta proteobacteria in the Proteobacteria phylum, and Anaeroplasmataceae family in the Tenericutes phylum were depleted in the b1/2-ARs KO chimera (Figure 2A). Analyses of gut microbial community structure by unweighted UniFrac principal coordinate analysis (PCoA) revealed two separate clusters, indicating dissimilarities in microbial community structure between the b1/2-ARs KO chimeras and the C57 controls (Figure 1C). Furthermore, a number of genera, including *Anaerotruncus, Coprococcus, Oscillibacter*, and *Desulfovibrio* were significantly reduced in b1/2-ARs KO chimeras compared to C57 control chimeras (Figure 2B). Therefore, deficiency in b1/2-ARs signaling in the BM led to an altered gut microbiota, characterized by a shift from Firmicutes to Bacteroidetes dominance, a reduction in the relative abundance of Proteobacteria, and diverse changes at the genus level. Interestingly, no significant dysbiosis was observed in the b1/2-ARs KO chimeras. By contrast, the shifts in gut microbial composition is considered beneficial due to the depletion of pathogenic bacteria and alteration in F/B ratio.

**Figure 3:**
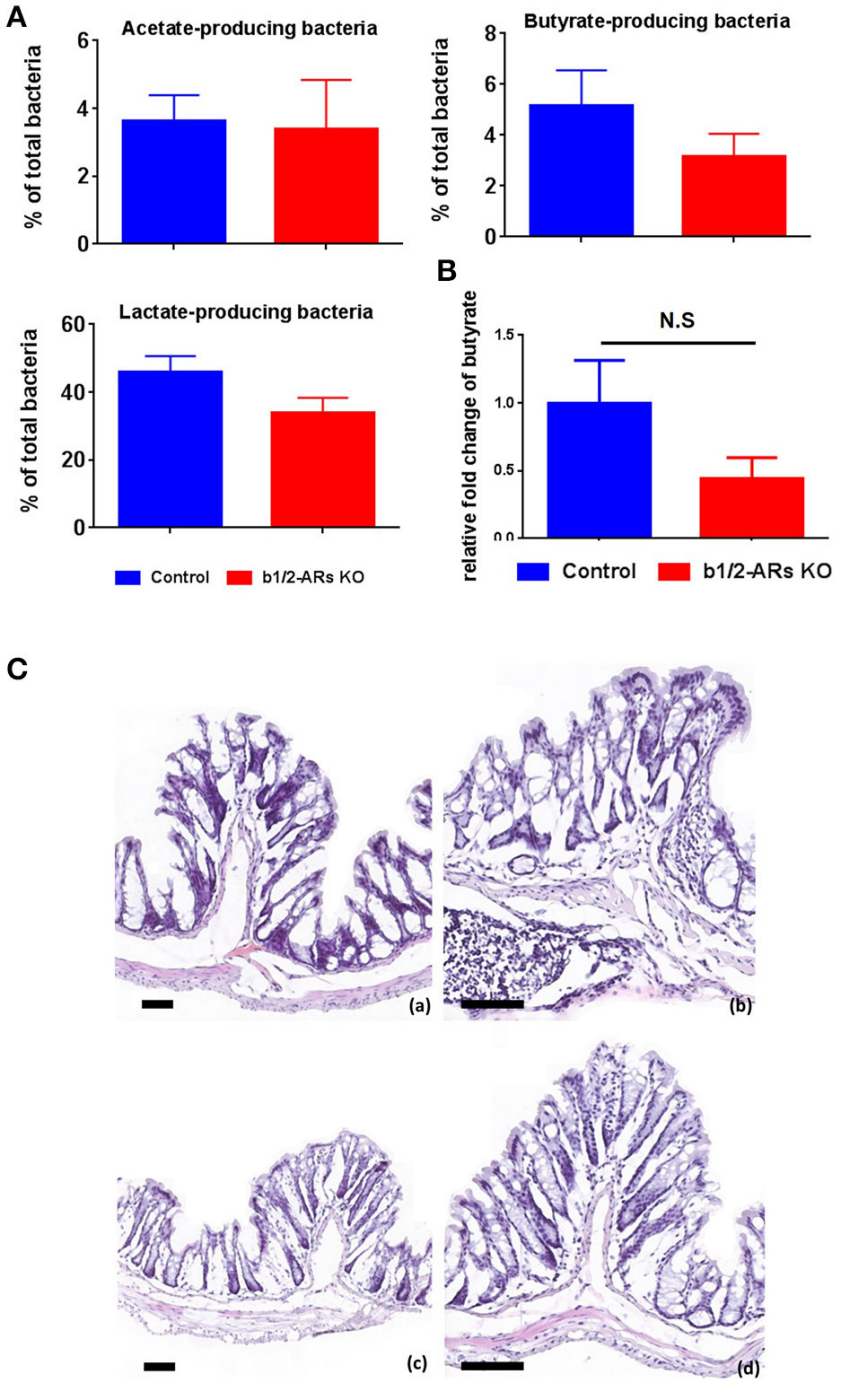
**Functional and histologic evaluation in the C57 and b1/2-ARs KO chimera. (A)** SCFA-producing bacteria and butyrate production in the feces. Percentages of acetate-, butyrate-, and lactate- producing bacteria in total gut microbiota. **(B)** No significant changes in the production of butyrate in the b1/2-ARs KO chimera. **(C)** Histology of C57 **(a,b)** and b1/2-ARs KO chimera **(c,d)** colons. (Frozen sections stained with hematoxylin and eosin, 100X **(a,c)** & 200X **(b,d)** magnification, all bars indicate 80 μm). No significant histopathologic lesions were detected in the colons of both C57 and b1/2-ARs KO chimera mice, and no qualitative differences were detected between the two groups.

Functionally, the end-products of anaerobic fermentation of the gut microbiota significantly contribute to the maintenance of intestinal and systemic homeostasis. Therefore, we grouped bacterial phylotypes at the genus level based on their primary fermented end-products (i.e., acetate, butyrate and lactate; Antharam et al., 2013; Yang et al., 2015). We did not observe significant alterations of total acetate, butyrate, and lactate-producing bacteria (Figure 3A) between C57 control and b1/2-ARs KO chimeras. However, hydrogen sulfide-producing bacteria, *Desulfovibrio*, was contracted in the b1/2-ARs KO chimeras (Figure 2B). HPLC analyses of fecal samples yielded no significant differences in butyrate levels between the KO and control chimeras, consistent with the bacterial analyses (Figure 3B). In the colon histological analysis, all sections from C57 and b1/2-ARs KO chimera animals were interpeted to be qualitatively normal, and no differences were detected between groups (Figure 3C). These indicate that the shift of gut microbial composition did not impact the major SCFA production and colon histology.

### Transcriptomic profiling identified three key transcripts associated with shifts in the gut microbiota

**Figure 4:**
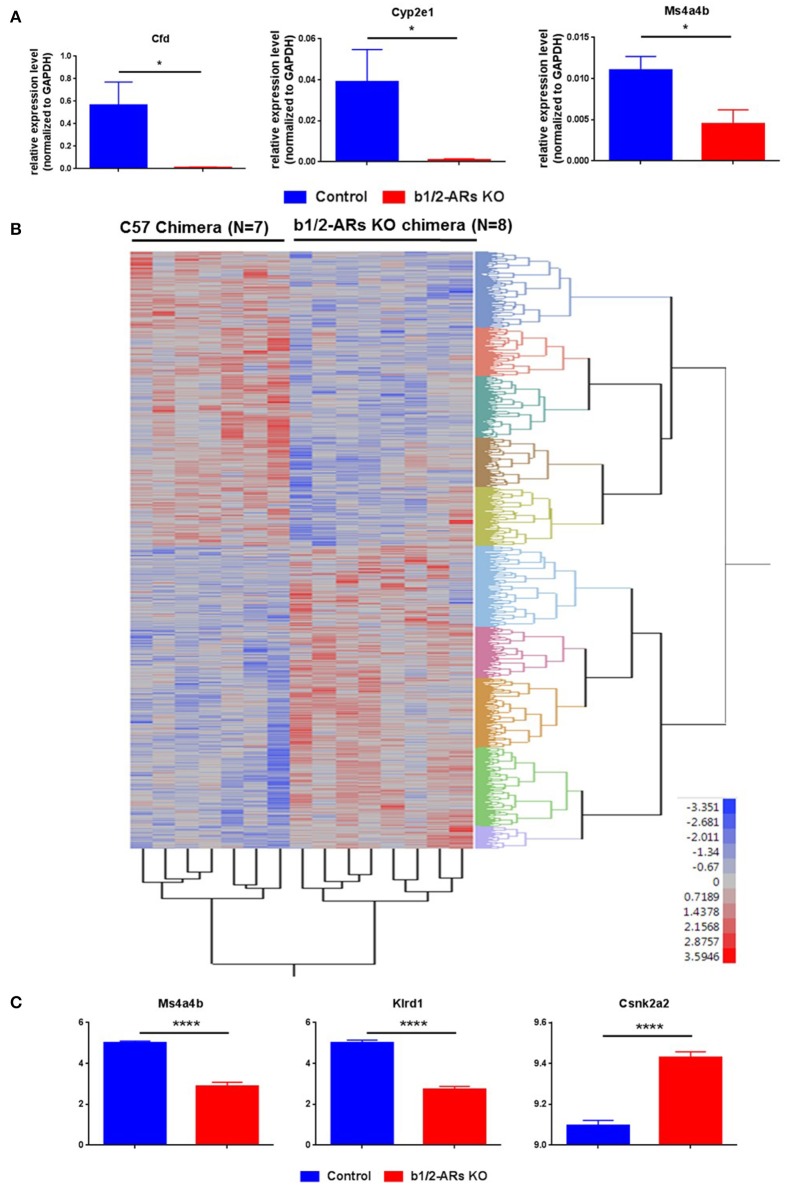
**Heatmap of differentially expressed probes (unadjusted, ***P*** < 0.05) between C57 and b1/2-ARs KO chimera. (A)** Validation of microarray data by real time PCR. Three genes were selected for validation of microarray analyses. Bar graph shows relative expression level of gene normalized to GAPDH expression. **(B)** There was a clear distinction between all individuals from each group, with two expression clades evident. **(C)** The three transcripts with the most significant differences between two groups in the microarray analyses are identified as Klrd1, Ms4a4b and Csnk2a2, presented in the bar graph. Two tailed unpaired *t*-test analysis indicates ^****^P < 0.0001. Adjusted *P*-values are 0.0042, 0.0085, and 0.0216, respectively. ^*^indicated unpaired *t*-test *P* < 0.05.

We have demonstrated that the loss of b1/2-ARs signaling in BM resulted in a shift in the gut microbiota. Due to the constant communication between intestine and the microbiota, we also measured the host colon transcriptome using a whole-genome microarray to determine global gene expression changes in the gut that may be involved in the microbiota shift. Based upon these data, we selected three abundantly expressed genes with significantly different expression levels (Appendix 1) between the C57 control and KO chimeras to validate the microarray data. Consistent with the microarray data, the expression levels of CFD, Cyp2e1, and Ms4a4b were decreased in the KO chimera mice (Figure 4A).

Subsequently, the microarray data were analyzed as described previously (Feswick et al., 2014; Ornostay et al., 2016). A vast number of differentially expressed probes in the distal colon of C57 vs. b1/2-ARs KO chimera mice were observed (*N* = 4,072, *P* < 0.05). All expression data are provided in Appendix 1. The two-way cluster is presented in Figure 4B to show the clustering of the individuals based on treatment. Thus, the cluster analyses using differentially expressed transcripts (unadjusted, *P* < 0.05) revealed that individual mouse from the two groups were separated based upon gene expression profiles. A core group of transcripts was uniquely different between the C57 control and the b1/2-ARs KO chimeras. Furthermore, microarray analyses identified three transcripts that showed significantly altered expression in the distal colon following an adjusted *P*-value (*FDR* = 5%): Killer Cell Lectin-Like Receptor Subfamily D, Member 1 (Klrd1), Membrane-Spanning 4-Domains, Subfamily A, Member 4A (Ms4a4b), and Casein Kinase 2, Alpha Prime Polypeptide (Csnk2a2). Ms4a4b is a CD20 homolog in T cells (Xu et al., 2010). Klrd1 is known as CD94 and is expressed on NK cells (Fang et al., 2011). Csnk2a2 is a protein kinase that promotes epithelial cell restitution (Song et al., 2000). Thus, these three differentially expressed genes are hypothesized to be important for both immune responses in the gut and communication with the microbiome. Herein, we observed significant upregulation of Csnk2a2 by 1.3-folds, while Klrd1 and Ms4a4b were downregulated ~4-fold in the distal colon of b1/2-ARs KO chimera mice (Figure 4C).

### Gene networks associated with intestinal processes and diseases are altered in b1/2-ARs KO chimera mice

**Figure 5:**
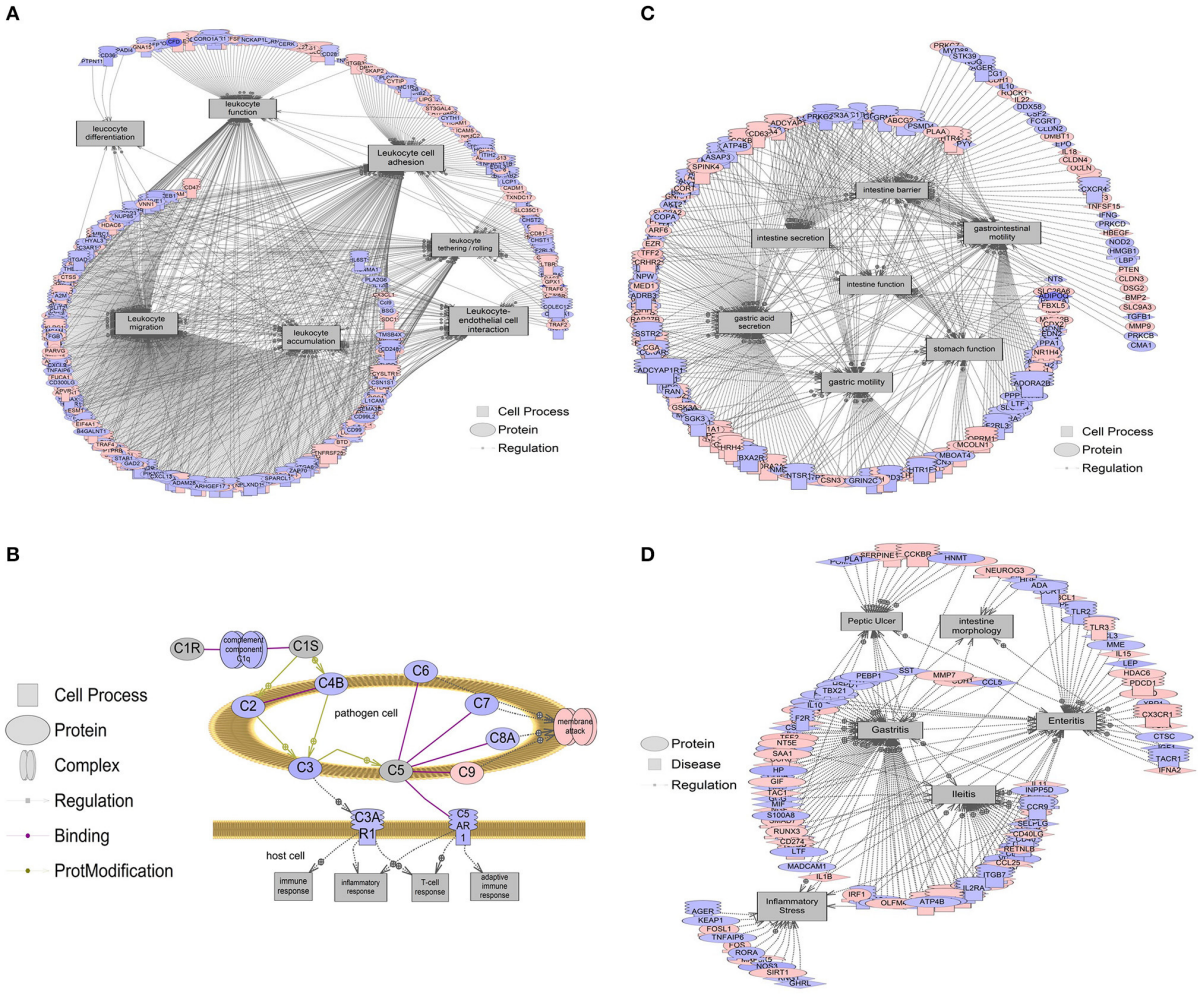
**Gene networks associated with intestinal processes and diseases**. Red indicates that relative mRNA levels are increased for the transcript, while blue indicates that relative mRNA levels are decreased for the transcript. The square entities refer to cell processes, the oval entities refer to proteins in general, triangles represent ligands, and receptors are those molecules embedded in the membrane with the mushroom cap. The arrows indicate direction of entity regulation. **(A)** Gene networks associated with leukocyte functions, including leukocyte adhesion, tethering/rolling, migration, accumulation, differentiation, and interaction with endothelial cells, were all altered. The mean fold change of the entire network is ~10% (1.1-fold). **(B)** Overall suppression in the classical complement system in the colon of b1/2-ARs KO chimera. The mean fold change of this gene network is ~21% decrease (1.21-fold). **(C)** Gene networks associated with intestine function, including barrier integrity, secretion, and motility were decreased by 6.5% (1.065-fold). **(D)** Gene networks associated with intestinal conditions including intestine morphology, enteritis, ileitis, and inflammatory stress are decreased by 7.5% (1.075-fold). Complete data are presented in Appendix 1 and 2. All genes within a pathway are located in Appendix 2.

We conducted further analyses of the host intestinal gene expression to identify specific host IS cellular processes that may be associated with the observed microbiota changes in the b1/2-ARs KO chimeras. Strikingly, the majority (262) of cell processes were downregulated at the transcriptome level, while only 16 gene networks were upregulated in the distal colon of the b1/2-ARs KO chimera (Appendix 2). Given that the three most affected genes, Ms4a4b, Klrd and Csnk2a2, are closely related to IS, we focused our analyses on cell processes involved in IS regulation. The cell processes most affected by the b1/2-ARs KO chimera included macrophage adhesion, NK cell proliferation and differentiation, mast cell chemotaxis, and leukocyte function, all showing significant downregulation in the distal colon at the transcriptome level (*P* < 0.01; Appendix 2). As an example, Figure 5A summarizes gene networks associated with leukocyte function (fold change by −1.103, *P* < 0.01). In addition, epithelial cell interaction (*P* < 0.001), permeability (*P* < 0.01), proliferation (*P* < 0.0001), and recycling (*P* < 0.05) were also downregulated (by ~8%, or 1.08-fold) in the distal colon of the b1/2-ARs KO chimera.

Furthermore, gene set enrichment analyses suggested that the classical complement pathway, which is an important part of the IS that contributes to phagocytosis and enhances the ability of antibodies to clear pathogens, was also suppressed in the distal colon of b1/2-ARs KO chimera (Figure 5B). By contrast, there were very few cell processes increased in the colon of b1/2-ARs KO chimera, and these included stromal cell function, cell-cell recognition, mucosal secretion, and processes associated with peripheral nerve blood flow. These processes increased at the level of the transcriptome by 10–20% (Fold change of 1.1–1.2; Appendix 2). Here we point out that this is the median fold change of the entire network, and networks are based on 100s of genes in some cases. Thus, fold changes for a network, when many genes are involved, are expected to be smaller compared to any single gene.

Due to the observed changes in the colon IS, sub-network enrichment analyses were performed in order to identify possible associations with diseases described in the literature (Borody et al., 2015; Alipour et al., 2016; Degruttola et al., 2016). This analysis revealed a significant association between the observed transcriptome changes in our b1/2-ARs KO chimera and diseases of the intestine function (*P* < 0.01), barrier integrity (*P* < 0.001), secretion (*P* < 0.05), and motility (*P* < 0.0001), all associated with 5–12% (1.05–1.12-fold) downregulation of gene networks in the b1/2-ARs KO chimera (Figure 5C). This suggests that intestinal function may be altered following the b1/2-ARs KO BM transplantation. Moreover, the transcriptome analysis revealed an overall downregulation of gene networks associated with diseases related to bowel inflammation (Figure 5D). For example, gene networks associated with ileitis were suppressed ~8% (*P* < 0.05), and included Cd36, Il4, Cd44, Cd4, and Cd40 (Appendix 2). Gene networks related to enteritis (*P* < 0.05) and intestinal morphology (*P* < 0.05) were also decreased at the transcriptome level in the colon of b1/2-ARs KO chimera. Additionally, a transcriptional network of inflammatory stress molecules was decreased by 6% (1.06-fold; *P* < 0.01). This network included genes such as Nos3, Il1b, Fos, Rora, Tlr4, and Fosl1, among others. A network related to inflammatory lesions (e.g., transcripts including Gf1, Ccl5, Cd8a, Il1b, Il1a, Il4, Il18, Cd4, Il15, Cx3cl1, Pparg, and Il6, among others) were also decreased by 8% (1.08-fold; *P* < 0.05). Lastly, obesity networks (e.g., transcripts including Retn, Lpl, Lep, Esr1, Lipc, Hsd11b1, Gpt, Lipe, Vldlr, and Il6, among others, fold change −1.042, *P* < 0.05) were also decreased by 4% (1.04-fold). All enriched disease networks are reported in Appendix 2.

### Decreased levels of specific ICs in the colon of b1/2-ARs KO chimera mice

**Figure 6:**
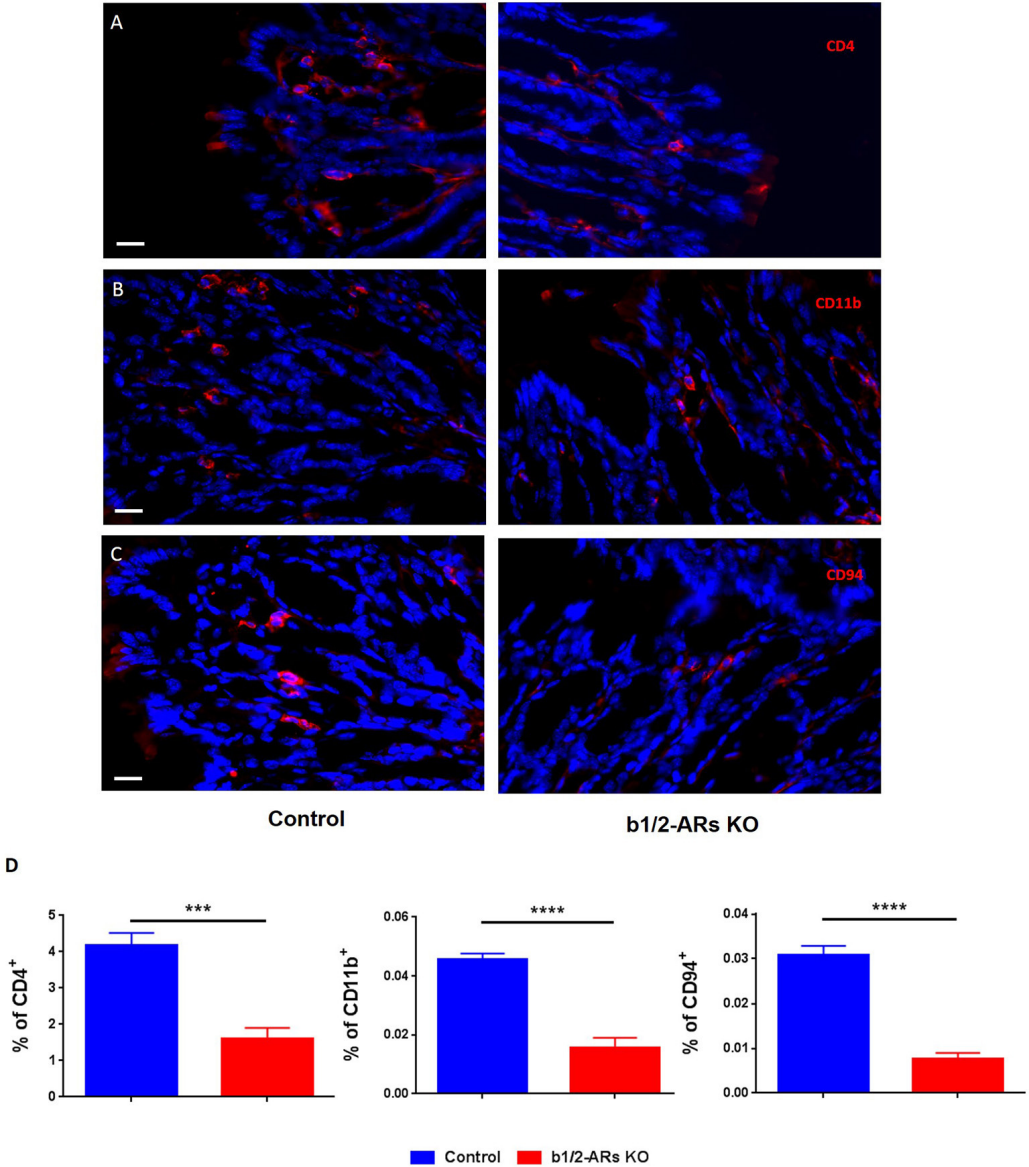
**Decreased infiltration of particular IC subsets in the colons of the b1/2-ARs KO chimera**. Significantly lower levels of CD4^+^ cells **(A)**, CD11b^+^ macrophages **(B)** and CD94^+^ NK cells **(C)** were observed in the colons of b1/2-ARs KO chimeras compared with C57 control chimeras. Quantified data were presented in the **(D)**. Blue indicates non-specific DAPI staining, while red shows CD4^−^, CD11b^−^, and CD94^−^ positive cells, respectively. Images were obtained at 400X magnification. All bars indicate 20 μm. Unpaired two tailed *t*-test showed ^***^P < 0.001 and ^****^P < 0.0001.

We report an overall suppression of the immune system in the gut of b1/2-ARs KO chimera. Since the suppressed transcripts, MS4a4b and Klrd1, are primarily expressed in T cells and NK cells, respectively, we proposed that the reduction in these gene transcripts in the colon would be associated with a decreased presence of infiltrating CD4^+^ T and NK cells in the colon of b1/2-ARs KO chimera. Thus, we employed immunofluorescence to quantify the relative frequencies of CD4^+^ T cells, CD11b^+^ macrophages, and CD94^+^ NK cells in the colon. Although there were no apparent qualitative histologic differences between the C57 and b1/2 ARs KO chimeras, we observed a reduced contribution of CD4^+^ T cells (by 61.8% or 1.62-fold; Figures 6A,D), CD11b^+^ macrophages (by 65.8%, Figures 6B,D), and CD94^+^ NK cells (by 75.2%, Figures 6C,D) within the colonic lamina propria of the b1/2-ARs KO chimera, in line with the observed reduction in immune responses at the transcriptome level.

## Discussion

An increasing number of diseases and conditions (i.e., hypertension, sleep apnea, anxiety) has been chacterized by both sympathetic dysfunction and gut microbial alterations (Tsioufis et al., 2011; Zubcevic et al., 2014). The present study was conducted to determine the association between IS, SNS and gut microbiota. Therefore, we utilized a novel BM chimera model to specifically induce deficiency in sympathetic regulation of the BM ICs. In this model, we present several novel findings: (i) We were able to establish a direct and novel link between sympathetic regulation of BM, local gut inflammatory networks, and gut microbiota, as ablation of b1/2-ARs in the BM caused significant shifts in fecal bacterial composition toward what is considered beneficial (i.e., comparable diversity and richness, reduced F/B ratio, and decreased Proteobacteria phylum; Mukhopadhya et al., 2012; Yang et al., 2015; Boulangé et al., 2016). Apart from a decrease in F/B ratio in the b1/2-ARs KO chimera, which contrasts the reportedly increased F/B ratio in obese and hypertensive patients and rodent models (Boulangé et al., 2016), we also observed a decrease in specific bacteria of the Proteobactarial phylum, of which many species are reportedly pathogenic (Batut et al., 2004; Mukhopadhya et al., 2012). (ii) Alteration in gut microbiota composition resulting from BM b1/2-ARs ablation is also associated with significant downregulation of inflammatory transcriptome networks in the colon, including leukocyte function, macrophage adhesion, NK cell proliferation and differentiation, leukocyte adhesion and migration, mast cell chemotaxis, and T-cell activation, among others. This is associated with marked reduction in specific subsets of ICs in the colon of the BM b1/2-ARs KO chimera. This suggests that modulation of BM beta-adrenergic signaling affects the gut IS. (iii) More specifically, we observed a significant downregulation in two IS-modulating genes in the colon of the BM chimera mice. Most interestingly, these genes are involved in regulation of T cell propagation (Ms4a4b) and NK cell activity (Klrd1), corresponding to the decreased frequency of T and NK cell infiltration to the colon of BM chimera mice. Furthermore, we observed an increase in Csnk2a2 steady state mRNA abundance, a protein kinase that promotes epithelial cell restitution, suggesting that improvement in colon epithelial proliferation may be associated with local immunosuppression in our chimera mice. This corroborates our results from the sub-network enrichment analyses, which shows an increase in the expression of a mucosal secretion network, a reported first line of IS defense in the gut (Deplancke and Gaskins, 2001; Kim and Khan, 2013), as well as significant decrease in gene transcriptome networks associated with inflammatory bowel disease, inflammatory stress and lesions, and obesity in the chimera mice. The latter may be particularly interesting, considering a recent publication suggesting a connection between expression of the Ms4a4 gene family in the gut and obesity (Pfalzer et al., 2016), and a well-reported association between obesity and increased SNS (Ando, 2014; Thorp and Schlaich, 2015).

Multiple physiological and immunological effects of SCFAs and lactate on the host's IS, gut function, and autonomic activity have previously been documented (Gao et al., 2009; Berni Canani et al., 2012; Lin et al., 2012; Furusawa et al., 2013; Al-Asmakh and Hedin, 2015; Bourassa et al., 2016). In our data, we did not observe significant changes in acetate, butyrate, and lactate-producing bacteria, and butyrate levels between the two groups. Consistent with this, no significant morphologic changes were observed in the colon of both groups, indicating that the observed microbiota shifts were not detrimental to the intestine.

Our transcriptome analysis revealed suppression of gene networks related to leukocyte function, intestine function, and intestinal diseases/conditions in the colon of KO chimera. In addition, we observed an overlapping reduction in Ms4a4b transcript in the BM (Ahmari et al., 2016) and colon of b1/2-ARs KO chimera, suggesting that expression of Ms4a4b may be directly regulated by the sympathetic drive to the BM. Ms4a4b is a CD20 homolog that modulates T cell proliferation and activation and plays a significant role in the enhancement of immune responses to antigen-induced signals (Howie et al., 2009; Xu et al., 2010). It also serves as a membrane adapter of glucocorticoid-induced tumor necrosis factor receptor (TNFR) family-related gene receptors, which are involved in potentiation of CD4 and CD8 T cell responses (Ronchetti et al., 2004). As the expression levels of both the BM and colon Ms4a4b transcripts are related to BM b1/2-ARs signaling, this suggests a novel convergence between the two pathways. However, further studies are required to determine whether Ms4a4b functions as a molecule link between the BM SNS and inflammatory responses in circulation as well as the gut. Similarly, Klrd1, also known as CD94, is a critical receptor involved in maintenance of NK cell function (Fang et al., 2011), the expression of which is also significantly reduced in the colon of b1/2-ARs KO chimera, and, again, follows the observed reduced infiltration of NK cells to the colon. The reduced mRNA levels of both Ms4a4b and Klrd1 in the b1/2-ARs KO chimera is also consistent with the significant suppression of systemic IS in the KO chimera, as discussed previously (Ahmari et al., 2016). On the other hand, expression of Csnk2a2, a protein kinase that promotes epithelial cell restitution and protects intestinal epithelial cells from inflammation-induced apoptosis (Koch et al., 2013), is increased in the colon of b1/2-ARs KO chimera, suggesting an improvement in epithelial function. Furthermore, our analyses points to an increase in networks associated with beneficial mucin secretion in the colon, as well as a reduction in gene networks associated with inflammatory bowel disease, inflammatory stress and lesions, and obesity in the chimera mice. Collectively, reduced infiltration of certain subsets of BM-derived ICs to the gut, as a result of the loss of b1/2-ARs signaling in BM, may be related to improved colonic epithelial homeostasis. Subsequently, the global immunosuppression in the gut may thus potentiate the beneficial shifts in the gut microbiota.

Considering the recent association between gut dysbiosis and hypertension (Pluznick et al., 2013; Jose and Raj, 2015; Mell et al., 2015; Yang et al., 2015), the observed beneficial shifts in the gut microbiota of the b1/2-ARs KO chimera may also play a significant role in reduction of BP previously reported in the same mouse model (Ahmari et al., 2016). Indeed, we demonstrated a decrease in the F/B ratio in the b1/2-ARs KO chimera, in contrast to the increased F/B ratios previously associated with obesity and hypertension (Yang et al., 2015; Boulangé et al., 2016). Furthermore, we observed a significant decrease in the Proteobacteria phylum in our KO chimera. Typically, the Proteobacteria phylum, characterized as the major group of Gram-negative bacteria, encompass a variety of potential pathogens that can potentiate activation of the host IS (Batut et al., 2004; Mukhopadhya et al., 2012). It is noteworthy that the gene set enrichment analyses identified downregulation of biological processes such as the “defense response to bacteria,” “defense response to Gram-positive bacteria,” “defense response to Gram-negative bacteria,” and “Gram-positive bacterial cell surface binding” in the b1/2-ARs KO chimera. This suggests a reciprocal host-microbiota interaction in our model, whereby, the primary effects on the microbiota are due to the initial BM-related IS suppression, while the secondary effects on the host may be exerted as a result of the changes in the composition of the microbiota.

We further observed a significant reduction in several potentially harmful bacteria in the colon of b1/2-ARs KO chimera. Though little is known about the role of *Thalassospira*, which belongs to the Rhodospirillaceae family, the presence of *Thalassospira* has been linked with diet-induced obesity (Clarke et al., 2013). Other species, such as those of the sulfide-reducing bacteria *Desulfovibrio*, have adverse effects on the host through the production of detrimental hydrogen sulfide, a reducing chemical that has potential genotoxic effects (Rowan et al., 2009). This may compromise gut barrier function (Roediger et al., 1993) and lead to host DNA damage (Attene-Ramos et al., 2007). The remarkably contracted portion of the *Desulfovibrionales* order in the b1/2-ARs KO chimera suggests a decrease in the production of hydrogen sulfide, although additional studies are required to confirm this. In the Firmicutes phylum, the *Anaerotruncus* genus is widely found in human stool samples, while it also contributes to 1% of total bacteria in the C57 control chimera. This genus contains only one species known as *Anaerotruncus colihominis*. Though its major end-products include acetate and butyrate, generally regarded as beneficial metabolites, the clinical investigation of *Anaerotruncus colihominis* demonstrated its critical role in bacteremia. In comparing intestinal lumen samples collected from colorectal cancer (CRC) patients and healthy individuals, *Anaerotuncus* is considered to be the key phylotype that contributes to the differences of intestinal lumen microbiota structure in CRC and healthy individuals (Lau et al., 2006; Chen et al., 2012). Thus, a contraction in this bacteria in our KO chimera may also be beneficial to the host. Another decreased bacteria genus in our b1/2-ARs KO chimera is *Coprococcus*, which is heavily associated with Crohn's disease and autism (Kaakoush et al., 2012; Kang et al., 2013; Gevers et al., 2014). *Coprococcus* also appears to show sensitivity to chronic stress, a condition with a well-established association with inflammation, SNS and gut microbiota shifts (Bailey et al., 2011). Thus, there appears to exist a role for *Coprococcus* in the regulation of intestinal and systemic immunity, in addition to its potential CNS effects. Furthermore, considering the reported association between stress and SNS (Goldstein, 1987; Teixeira et al., 2015), the observed contraction of *Coprococcus* may be partly related to reduced SNS effects on the BM in the b1/2-ARs KO chimera. We also observed a potentially beneficial contraction in the *Oscillibacter* in the KO chimera, a bacterial genus implicated in body weight gain in high fat diet-fed mice (Lam et al., 2012). The relative abundance of *Oscillibacter* also shows a negative correlation with tight junction protein expression and intestinal trans-epithelial resistance, indicating that higher relative abundance may be detrimental for the gut barrier. Interestingly, *Oscillibacter* may also impact the CNS, as it is linked with depression (Naseribafrouei et al., 2014; Jiang et al., 2015).

In summary, this study specifically depletes the sympathetic regulation on the BM to demonstrate the interplay between the SNS and IS in maintenance of gut and microbiota homeostasis. Depletion of this sympathetic regulation on BM leads to beneficial shifts in gut microbiota associated with gut immune suppression. Thus, regulation of the efferent sympathetic arm of the ANS may be therapeutically relevant in immune disorders that are associated with gut dysbiosis. Co-administration of already available medications such as beta-blockers may prove beneficial in these conditions. In addition, we identify changes in specific molecules in the gut that may be involved in mediating host-microbiota interaction and thus may present novel therapeutic targets for immune disorders presenting in the gut.

## Availability of data and materials

Raw microarray data have been deposited into the NCBI Gene Expression Omnibus (GEO) database (Series GSE71632, GPL13912). Raw 16S RNA amplicon sequencing data have been deposited into the NCBI Sequence Read Archive (SRA) database (SRA Submission SRP094562, BioProject PRJNA355172, BioSample SAMN06065847-SAMN06065856).

## Author contributions

TY designed the experiments, performed the 16S rRNA amplicon sequencing, analyzed sequencing data, performed IHC staining, interpreted the data, and contributed to writing of the manuscript. NA designed the experiments and collected tissues for RNA transcriptomics. JS performed RNA transcriptomics. TR performed quantitative PCR. RA quantified IHC staining. KP quantified IHC staining. KM performed HPLC and interpreted the data. WM performed animal experiments and IHC examination. JO performed animal experiments, and contributed to manuscript writing. GK evaluated colon histology and contributed to writing of the manuscript. EL performed the 16S rRNA gene amplicon sequencing bioinformatic analysis. GW interpreted the 16S rRNA gene amplicon sequencing data. TV interpreted HPLC data. MR contributed to manuscript writing. CM designed the experiments, interpreted transcriptomic analyses and contributed to manuscript writing. JZ designed the experiments, and wrote the manuscript.

## Funding

Supported by AHA grant 14SDG18300010 to JZ and University of Florida College of Veterinary Medicine (UFCVM) Start Up Funds to JZ and CM.

### Conflict of interest statement

The authors declare that the research was conducted in the absence of any commercial or financial relationships that could be construed as a potential conflict of interest.

## Abbreviations

IS: Immune System
IC: Immune Cell
SNS: Sympathetic Nervous System
a/b-ARs: alpha/beta-Adrenergic Receptors
BM: Bone Marrow
BP: Blood Pressure
LDA: Linear Discriminant Analysis
LEfSe: LDA along with Effect Size measurements
SCFA: Short Chain Fatty Acid
HPLC: High Performance Liquid Chromatography
PCoA: Principal Coordinate Analyses
SNEA: Sub-Network Enrichment Analysis
Ms4a4b: Membrane-Spanning 4-Domains Subfamily A, Member 4A
Klrd1: Killer Cell Lectin-Like Receptor Subfamily D, Member 1
Csnk2a2: Casein Kinase 2, Alpha Prime Polypeptide
TNFR: Tumor Necrosis Factor Receptor
CRC: Colorectal Cancer.
